# Readability of Chatbot Responses in Prostate Cancer and Urological Care: Objective Metrics Versus Patient Perceptions

**DOI:** 10.3390/curroncol32100582

**Published:** 2025-10-19

**Authors:** Lasse Maywald, Lisa Nguyen, Jana Theres Winterstein, Martin Joachim Hetz, Maurin Helen Mangold, Luisa Vivienne Renner, Titus Josef Brinker, Frederik Wessels, Nicolas Carl

**Affiliations:** 1 Department of Urology, University Medical Center Mannheim, 68167 Mannheim, Germany; 2 Medical Faculty Mannheim, Ruprecht-Karls University of Heidelberg, 68167 Mannheim, Germany; 3 Division of Digital Prevention, Diagnostics and Therapy Guidance, German Cancer Research Center (DKFZ), 69120 Heidelberg, Germany; 4 Medical Faculty, Ruprecht-Karls University of Heidelberg, 69120 Heidelberg, Germany

**Keywords:** large language models, GPT-4, chatbots, patient education, readability, health literacy, urology, artificial intelligence, urooncology

## Abstract

**Simple Summary:**

Large language models (LLMs) are being used more often in healthcare, but we still know little about how well they help patients understand medical information. Until now, no study has asked patients directly how clear they find information generated by LLMs. To address this, we conducted a survey comparing how readable chatbot responses were with how easy patients said they were to understand. Knowledge retention was not assessed. Urology patients used a voice-based chatbot to ask medical questions during regular clinic visits. We analyzed transcripts from 231 conversations. While chatbot answers were written at a level higher than typically recommended for patient education, most patients still found the information understandable. We believe this may be due to how patients received the information, by both reading and listening, which might make it easier to understand. These findings suggest the benefit of multimodal designs in health tools to meet patients’ diverse needs.

**Abstract:**

Large language models (LLMs) are increasingly explored as chatbots for patient education, including applications in urooncology. Since only 12% of adults have proficient health literacy and most patient information materials exceed recommended reading levels, improving readability is crucial. Although LLMs could potentially increase the readability of medical information, evidence is mixed, underscoring the need to assess chatbot outputs in clinical settings. Therefore, this study evaluates the measured and perceived readability of chatbot responses in speech-based interactions with urological patients. Urological patients engaged in unscripted conversations with a GPT-4-based chatbot. Transcripts were analyzed using three readability indices: Flesch–Reading-Ease (FRE), Lesbarkeitsindex (LIX) and Wiener-Sachtextformel (WSF). Perceived readability was assessed using a survey covering technical language, clarity and explainability. Associations between measured and perceived readability were analyzed. Knowledge retention was not assessed in this study. A total of 231 conversations were evaluated. The most frequently addressed topics were prostate cancer (22.5%), robotic-assisted prostatectomy (19.9%) and follow-up (18.6%). Objectively, responses were classified as difficult to read (FRE 43.1 ± 9.1; LIX 52.8 ± 6.2; WSF 11.2 ± 1.6). In contrast, perceived readability was rated highly for technical language, clarity and explainability (83–90%). Correlation analyses revealed no association between objective and perceived readability. Chatbot responses were objectively written at a difficult reading level, exceeding recommendations for optimized health literacy. Nevertheless, most patients perceived the information as clear and understandable. This discrepancy suggests that perceived comprehensibility is influenced by factors beyond measurable linguistic complexity.

## 1. Introduction

A central challenge in patient education is readability. Only about 12% of adults demonstrate proficient health literacy, while most medical information materials are written at a complexity exceeding readability level recommended by the National Institutes of Health (NIH) and American Medical Association (AMA), i.e., sixth or eighth grade reading levels [1]. Individuals with lower educational attainment face disproportionate barriers in comprehending complex health information, which may contribute to inequities in shared decision-making [2]. This issue is particularly critical in urooncology, where high clarity in communication is demanded by the rapid emergence of novel therapeutic evidence, surveillance protocols and the profound consequences that miscommunication could carry in cancer care. Although recent work suggests that LLMs can simplify complex language and thereby enhance the readability of patient education materials, evidence from real-world clinical settings is heterogeneous [3].

Studies consistently show that patient education materials (PEMs) for urological cancers exceed recommended reading level thresholds. The Flesch–Kincaid Reading Ease (FRE) is a readability metric ranging from 0 to 100 that estimates the school grade level required to understand a given text, with higher scores indicating simpler text and improved accessibility [4]. For example, a score between 70 and 80 is equivalent to school grade level 8. Rodler et al. demonstrated that most urooncological PEMs fail to reach an FRE-Score ≥ 70 [2]. Similarly, Pruthi et al. reported average readability levels of grade 11.7 for online urooncology resources [5]. These findings underscore a substantial gap between current and recommended standards in PEMs.

Large language models (LLMs) in the form of chatbots are increasingly investigated as conversational tools for patient education, including applications in urooncology [6]. By allowing natural language queries and generating contextualized answers, LLMs are positioned to complement or even replace traditional search engines as a primary source of medical information for patients [7,8]. Moreover, LLMs are dynamic and can be adapted to the specific needs of the user when prompted, offering the potential for personalized and accurate health communication. Recent evaluations suggest that patients prefer chatbots over search engines for user-friendliness and quality of information [9].

However, the challenge of subpar readability extends to LLM-generated PEMs. Prior work has assessed the quality and readability of such materials in controlled or in-silico settings. For example, Hershenhouse et al. found that LLM outputs on prostate cancer often exceeded typical comprehension levels [10,11]. This challenge can be addressed as exemplified by two recent studies. Ganjavi et al. recently presented the first randomized controlled trial showing that LLMs can simplify scientific abstracts, and Rodler et al. reported that GPT-4 can significantly improve the objective readability of PEMs with the use of evidence-based reference knowledge and prompt engineering [12,13].

Building on this line of research, the Interaction of Patients with Large Language Models (IPaLLM) study was designed to evaluate chatbot use in a clinical setting [9,14,15]. Here, urological patients engaged in unscripted conversations with a speech-based GPT-4 chatbot during routine care visits. The aim of this study is to assess the correlation between objective and perceived readability of outputs generated by a GPT-4-based LLM in response to patient inquiries. Patients interacted with a chatbot in unscripted conversations to obtain medical information. In addition, we report descriptive data on the conversation transcripts and demographic characteristics of the participants. We hypothesize that the conversational, speech-to-speech delivery of information by the chatbot may enhance perceived readability, even when the objectively measured readability levels remain comparatively complex.

## 2. Materials and Methods

### 2.1. Study Design and Participants

This explorative analysis was conducted using data collected as part of the prospective clinical trial IPaLLM (Interaction of Patients with Large Language Models), officially registered with the German Clinical Trial Registry (DRKS-ID: 00034906, registered on 16 of August 2024). The trial prospectively enrolled adult patients attending urological consultation at the University Medical Center Mannheim, excluding those with cognitive impairments or psychiatric conditions. Eligible participants engaged in conversation-like interactions with a speech-to-speech LLM-powered chatbot (GPT-4, OpenAI) and subsequently completed a survey. This work shares a unified study design and methodology with previously published parts of the clinical trial, which examined patients’ confidence in LLM capabilities as well as their perceptions of usefulness, quality, and empathy when interacting with a chatbot for medical information-seeking [9,14,15].

The current study specifically investigates chatbot–patient transcripts with regard to objective readability metrics and perceived readability, thus extending our prior research that predominantly assessed the patient’s perspective. This evaluation was approved by the institutional ethics review board of the Medical Faculty Mannheim, Ruprecht-Karls University of Heidelberg (proposal number: 2023-687, approval date: 27 February 2024) and adhered to the principles of the Declaration of Helsinki.

### 2.2. Study Procedure

After providing informed consent, participants received an introduction to LLMs and then interacted with a GPT-4 powered chatbot on a tablet, asking any medical question relevant to their current situation. No specific prompt engineering was undertaken to increase readability. The chatbot session was followed by a structured post-interventional survey, including the assessment of the perception of output readability. Demographic data, including age and educational attainment, were non-mandatory items collected at the end of the procedure. This analysis evaluated objective readability (FRE/LIX/WSF) and patient-reported perceived readability. Clinical correctness (relative to guideline statements or local protocols) was not assessed. Conversations were conducted immediately prior to the clinical encounter, allowing the treating physician to review and correct information as needed.

### 2.3. Questionnaire Development

The survey instrument used in this study was developed based on the existing literature and refined through six semi-structured expert interviews, comprising both patients and board-certified urologists. The resulting questionnaire was tested in a pilot study involving 20 medical students. The survey assessed perceived readability across four items: “understandability of technical language” and “content” as well as “clarity” and “explanation sufficiency”. Agreement for items was captured using a Likert scale ranging from 1 (strongly disagree) to 5 (strongly agree), survey items are displayed in Table 1.

### 2.4. Conversation Analysis

First, transcripts of chatbot–patient conversations were evaluated descriptively. Conversations were classified by discussed topics (e.g., disease, procedure and general medical theme if applicable) as well as analyzed for total word and sentence count. Secondly, readability was assessed objectively using three established indices: Flesch Reading Ease (FRE) [4], Lesbarkeitsindex (LIX) [16], and Wiener Sachtextformel (WSF) [17]. Given that all interviews were conducted in German, the adapted formula FRE_German_ for the German language was employed [18]. In addition, the LIX and WSF readability indices were selected as complementary measures. Their results were later compared to those obtained from the FRE_German_ analysis.

### 2.5. Readability Indices

This section outlines the calculation formulas of the readability indices applied in this study.

The FRE_German_ is calculated as FRE_German_ = 180 − ASL − (58.5 × ASW), where ASL denotes the average sentence length in words and ASW the average number of syllables per word [18]. Higher values indicate greater ease of reading, with scores above 80 reflecting easy-to-read texts and values below 30 denoting very difficult academic-level text. Throughout this manuscript, FRE_German_ is hereafter referred to as FRE. Both NIH and AMA recommend an FRE of ≥70 for PEMs.

The LIX formula is LIX = (A/B) + (C × 100/A), where A represents the total number of words, B the number of sentences, and C the number of long words with more than six letters [16]. Here, higher scores correspond to more complex texts, with values above 60 considered very difficult, between 40 and 50 easy, and below 40 very easy.

The WSF is defined as WSF1 = 0.1935 × MS + 0.1672 × SL + 0.1297 × IW − 0.0327 × ES − 0.875, where MS denotes the percentage of words with three or more syllables, SL the average sentence length in words, IW the percentage of words longer than six letters, and ES the percentage of monosyllabic words [17]. As with LIX, higher WSF1 values indicate greater complexity, with scores between 7 and 10 representing well understandable content and values above 12 considered difficult to very difficult.

### 2.6. Statistical Analysis

We first conducted a descriptive evaluation of chatbot–patient conversations.

Readability of chatbot outputs was quantified using FRE, LIX and WSF1. To explore differences in readability across conversation topics (disease, procedure, theme), we applied Kruskal–Wallis rank sum tests. This non-parametric approach was selected due to the non-normal distribution of readability scores and unequal group sizes. Results are reported with corresponding effect measures, 95% confidence intervals (95% CIs) and *p*-values.

Perceived readability was assessed using four Likert-scale items. Although single items are ordinal, aggregation into a composite score with good internal consistency (Cronbach’s α = 0.75) allows treatment as an interval-scaled variable. Also, an exploratory factor analysis (EFA) was conducted to further examine the dimensionality of the scale.

Correlations between objective readability indices and perceived readability were estimated using Spearman’s rank correlation coefficients. These analyses were defined a priori, and results are reported with ρ-coefficient, 95% CIs and *p*-values.

Lastly, we explored the potential influence of patient characteristics on perceived readability. A multiple linear regression model was fitted including the composite perceived readability score as the dependent variable. Age and educational level were included as predictors, as they were considered potentially influential on health literacy. Regression results are reported with coefficients (β), 95% CIs and *p*-values.

All statistical analyses were performed using RStudio (Version 2025.05.0 + 496, Posit Software, PBC, Boston, MA, USA). A significance level of 0.05 was applied throughout. Only the main results are reported within the manuscript, for a detailed view of the comprehensive results we refer to the Supplementary Materials.

## 3. Results

### 3.1. Conversation Content Analysis

To contextualize patient–chatbot interactions beyond readability metrics, we performed a qualitative analysis of the conversation content. A total of 231 chatbot–patient conversations were analyzed and categorized according to the discussed topics: disease, procedure, and overarching theme.

Regarding disease-related topics, the most frequently discussed conditions included prostate cancer (52/231; 22.5%), urothelial cancer (33/231; 14.3%), and suspicious PSA elevation (27/231; 11.7%). Procedural discussions were led by robotic-assisted radical prostatectomy (RARP, 46/231; 19.9%), prostate biopsy (27/231; 11.7%), and Holmium Laser Enucleation of the Prostate (HoLEP, 24/231; 10.4%). Conversations also covered a diverse range of interventions, including kidney cancer surgery (17/231; 7.4%), lithotripsy procedures (13/231; 5.6%) and radical cystectomy with urinary diversion (12/231; 5.2%). However, 44 of 231 (19.1%) transcripts were not applicable to a specific procedure, because they were either focused on a specific disease or on overarching medical themes (e.g., follow-up, prognosis).

**Figure 1:**
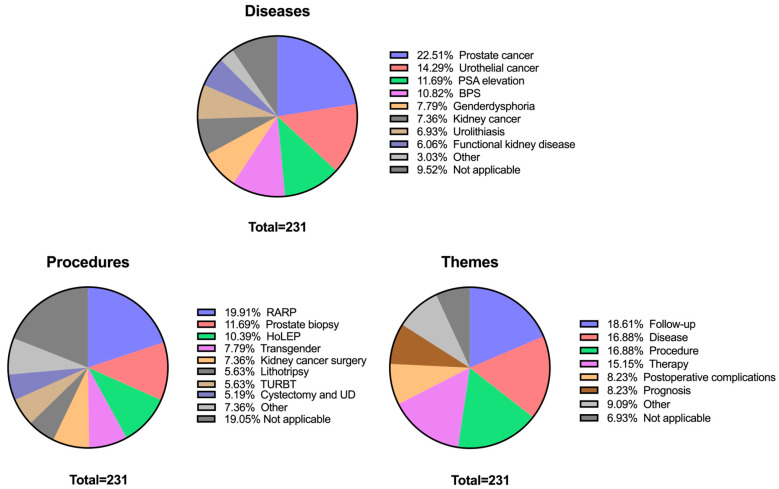
Distribution of chatbot conversations across three topic categories: diseases, procedures, and themes. Each pie chart shows the relative frequency of discussed topics within the sample (*N* = 231). The label “Not applicable (NA)” was recurrent across categories, reflecting that some patient queries focused solely on procedures without reference to a disease, or vice versa. Diseases, procedures, and themes with a frequency below 5% were grouped together and listed as “Other.” Specifically, this included: Diseases: Urethral stenosis (1.73%), Testicular cancer (0.87%), and Penile cancer (0.45%). Procedures: DJ replacement (3.9%), Chemotherapy (0.87%), Orchidectomy (0.43%), Urethral reconstruction (1.3%), Pyeloplasty (0.43%), and Partial penectomy (0.43%). Themes: Behavior (2.6%), Diagnostics (2.6%), Prevention (1.73%), Medication (1.3%), and Psychosocial (0.87%).

When categorized into overarching themes, questions about follow-up (43/231; 18.6%), disease biology (39/231; 16.9%) and procedural steps (39/231; 16.9%) dominated. General therapy related questions constituted 35 of 231 (15.2%) of conversations, while postoperative complications (19/231; 8.2%) and prognostic concerns (19/231; 8.2%) were also recurrent themes. A comprehensive overview of discussed topics is provided in Figure 1.

### 3.2. Conversation Readability Analysis

To complement the qualitative assessment of conversational topics, we next examined quantitative transcript metrics, including word and sentence counts as well as readability indices (FRE, LIX, WSF). This analysis was aimed at evaluating both pooled and grouped (by topics) readability of chatbot outputs.

Across all transcripts, the average language was classified as difficult according to the FRE (43.07; SD 9.06), while LIX indicated a difficult level (52.82; SD 6.17), and WSF corresponded to an upper-secondary reading level (11.20; SD 1.63). On average, responses contained 175 words (SD 54) distributed across approximately 15 sentences (mean 14.74; SD 6.52).

Readability indices (FRE, LIX, WSF) did not differ meaningfully with respect to disease, procedure or overarching theme discussed (all *p* ≥ 0.5; all ε^2^ small with 95% CIs spanning near zero). A single nominally significant finding (procedure–LIX, *p* = 0.031, ε^2^ = 0.05) would not remain significant after correcting for multiple comparisons. Word and sentence counts differed across thematic categories (words: *p* = 0.007; sentences: *p* = 0.004), yet effect sizes are not tabulated, since exploratory analyses revealed no meaningful or robust group differences between topic categories and readability indices. Detailed results of readability indices stratified by topic categories are provided in Supplementary Tables S3–S5.

### 3.3. Perceived Readability

**Figure 2:**
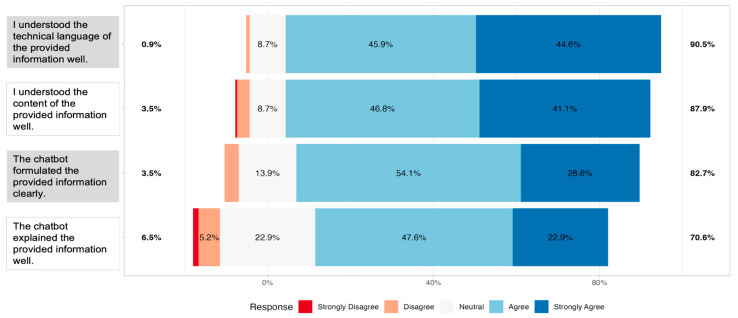
Centered distribution of responses of *n* = 231 participants on a 5-point Likert scale for the four readability items (H1: understanding of technical language; H2: understanding of content; E2: clarity of formulation; E4: quality of explanation). Bars represent the proportion of participants selecting each response option, with colors indicating the level of agreement from “Strongly Disagree” to “Strongly Agree” Percentages on the sides reflect the combined share of respondents who agreed/disagreed or strongly agreed/strongly disagreed with each item.

Next, we examined patients’ subjective evaluations of chatbot output readability. Survey responses showed that most participants perceived the outputs as readable across all four items. For the statement on understanding the technical language of the provided information, 90.5% of respondents agreed or strongly agreed, with only 1% reporting strong disagreement. Similarly, 87.9% agreed or strongly agreed to have “understood the content of the provided information well”. The statement “the chatbot formulated the provided information clearly” was endorsed positively by 82.7% of participants. The lowest level of agreement was observed for the item “The chatbot explained the provided information well”; however, 70.6% still agreed or strongly agreed (Figure 2).

Together, these findings demonstrate that, despite objectively difficult readability levels, patients largely perceived the responses of the chatbot as clear, understandable and explanations as appropriate.

An exploratory factor analysis (maximum likelihood extraction, oblimin rotation) was conducted on the four readability items. Internal consistency was acceptable (Cronbach’s α = 0.75). A two-factor solution accounted for 77% of the variance. Factor 1 (ML1) was defined by the H-items, with loadings of 0.80 (H1) and 1.00 (H2). Factor 2 (ML2) was defined by the E-items, with loadings of 1.00 (E2) and 0.60 (E4). The two factors were moderately correlated (r = 0.30).

### 3.4. Correlation Analysis

Strong positive correlations were observed between the LIX and WSF readability indices (ρ = 0.88; *p* < 0.001; 95%CI = 0.82–0.90), while both showed strong negative correlations with the FRE index (FRE vs. LIX: ρ = −0.82; *p* < 0.001; 95%CI = −0.85–0.74; FRE vs. WSF: ρ = −0.92; *p* < 0.001; 95%CI = −0.92−0.87), reflecting the inverse scaling of these measures. Word count correlated moderately with the number of sentences (ρ = 0.57; *p* < 0.001; 95%CI = 0.46–0.64), but only weakly with readability metrics. In total, the German-specific indices LIX and WSF correlated strongly with FRE, indicating internal validity of the applied metrics.

**Figure 3:**
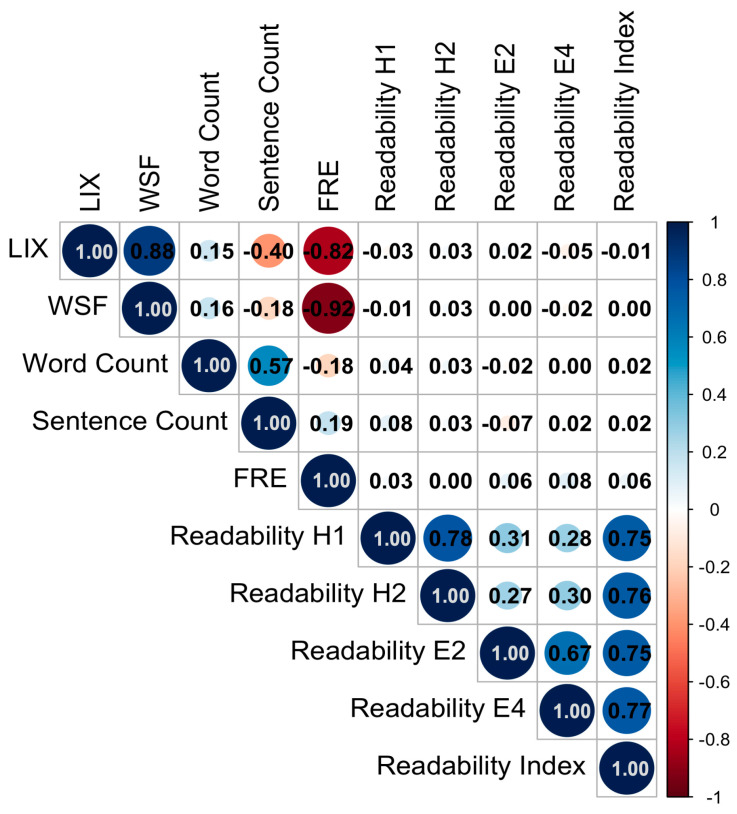
Correlation plot of objective readability measures (LIX, WSF, word count, sentence count, FRE) and patients’ subjective readability evaluations (H1: understanding of technical language; H2: understanding of content; E2: clarity of formulation; E4: quality of explanation). Subjective readability items were highly intercorrelated and showed only weak associations with objective readability metrics.

Interestingly, no notable correlations were observed between objective readability indices (FRE, LIX, WSF) and perceived readability items directly (see Figure 3).

### 3.5. Demographic

In total 230 of 231 participants provided demographic data. Participants were recruited primarily during pre-admission consultations for upcoming urological interventions, potentially yielding a sample that was more digitally engaged than typical outpatient populations. The mean age was 60.97 years (SD 15.91), most participants were senior or elderly (190/230, 83%). Over the past year, the majority reported frequent healthcare contact: 65% (148/229) visited a physician more than five times and 35% (80/229) reported 1–5 visits. Most respondents had used the internet for medical information (195/229, 85%), evenly distributed across age strata. More than half used the internet daily for medical questions (119/229, 52%), while 33% used it weekly and 15% monthly. Google was the predominant information source (181/214, 85%), followed by Wikipedia and official patient forums (each 8/214, 3.7%); other sources were rare. Prior use of an LLM for medical questions was reported by only 9.0% (20/222), with a clear age gradient (young adults 15%, adults 25%, seniors 8.5%, elderly 5.4%; *p* = 0.029). Among LLM users, ChatGPT was most frequently named (19/22, 86%). The sample was predominantly male (167/226, 74%), representing the distribution of a real-world urological cohort. Educational attainment was reported by 193 of the 231 participants. Among these, 26 patients (13%) were classified as ISCED level 1, 68 (35%) as ISCED level 2, 31 (16%) as ISCED level 3 and 68 (35%) as ISCED level 4. Educational attainment was unknown for 37 patients (16%). Patients were distributed ranging from vocational training to higher academic degrees.

Furthermore, the potential influence of demographic factors (age and educational level) on perceived readability outcomes was explored. Neither age nor educational level showed statistically significant associations with perceived readability outcomes (all *p* > 0.15). The effect estimates were small, and confidence intervals crossed zero throughout, indicating no meaningful relationship. Thus, perceived readability appeared independent of participants’ age and educational attainment within the current evaluation. Tabulated results of the regression are displayed in Supplementary Table S6.

## 4. Discussion

The present study aimed to evaluate how patients experience the readability of chatbot-generated health information in urological care. By analyzing 231 transcripts from a speech-mediated GPT-4 chatbot, we assessed both: objective readability using established indices, and patients’ perceived readability through reported outcomes. We found that although chatbot responses were objectively written at a higher-than-recommended level, patients generally perceived them as understandable.

This work extends prior research which primarily surveyed patient perceptions of LLMs. Earlier analyses focused on user-reported outcomes such as informational quality, user-friendliness, empathy, self-disclosure and trust in medical decision-making when comparing chatbots, search engines, and physicians [9,14,15]. In contrast, the present analysis examines the conversations themselves, enabling the first direct comparison between objective readability metrics and patients’ subjective evaluations.

Notably, participants rated the information as highly clear and understandable despite the objectively complex reading levels. Perceived readability did not vary with age or education, and there was no association between topic complexity and subjective clarity. Several factors may explain this phenomenon. First, participants interacted with the chatbot through speech while concurrently reading the transcribed answers. This multimodal information intake may have enhanced comprehension by engaging multiple sensory channels and allowing patients to process content at their own pace. A meta-analysis of 30 studies found an overall benefit of reading while listening versus reading only, supporting the plausibility of our multimodal explanation [19]. Second, the conversational format permitted patients to steer discussions toward personally relevant questions. Such dynamic, interest-driven engagement likely increased motivation and cognitive focus, making lengthy explanations easier to follow. Third, the answers of the chatbot were verbose. Although verbosity inflates readability scores, elaboration may provide additional explanations. Together, we showed that there is a dissonance between readability indices and reported readability suggesting that readability indices may not fully capture the cognitive processes involved in interactive, speech-based learning.

A further aspect to consider is the potential influence of the Dunning–Kruger effect on our subjective readability ratings. The Dunning–Kruger effect is a cognitive bias whereby individuals with limited knowledge or skill overestimate their own competence [20]. In the context of health literacy, research has shown that respondents with objectively low health literacy reported as much or more confidence in their health knowledge than those with higher literacy, despite engaging in more problematic health behaviors and failing to recognize their informational deficits [21]. Moreover, studies highlight that health literacy is generally low in the population and that people are unrealistically optimistic about their health risks [22]. This effect appears to be particularly pronounced among male individuals [23], who constitute approximately two-thirds of the urological patient population. Applied to our cohort, these findings raise the possibility that some participants, particularly those with lower health literacy or who are male, may have overestimated their understanding of the explanation of the chatbot.

Additionally, response biases such as social desirability may further inflate perceived readability. Social desirability bias refers to a pervasive response bias in which individuals tailor their answers to align with perceived social norms or to present themselves in a favorable light. Acquiescent response sets have also been shown to upwardly bias satisfaction scores when items are positively worded [24]. Experimental evidence from patient satisfaction surveys demonstrates that positively framed statements yield markedly higher satisfaction than the same items presented negatively [25], while demographic factors like older age and lower education are associated with higher satisfaction regardless of content [26]. These observations indicate that subjective readability ratings may partly reflect response tendencies and social expectations rather than genuine comprehension.

LLM outputs were objectively demanding, yet showed no significant differences in readability across diseases, procedures, or overarching themes. Procedure-related questions ranged from simple interventions such as prostate biopsy to complex procedures like radical cystectomy, yet objective readability metrics remained uniformly high across categories. The absence of statistically significant differences in FRE, LIX or WSF between simple and complex topics suggests that the model produces text at a relatively stable level of linguistic complexity. That level consistently exceeds recommended sixth-grade thresholds, mirroring findings in other specialties where unmodified ChatGPT outputs for otosclerosis, chemotherapy cardiotoxicity or prostate cancer information required college-level comprehension [11,27,28]. In a study by Thia et al., chatbot-generated urological explanations demonstrated poor Flesch–Kincaid performance, but readability markedly improved once the prompt was adapted to generate content suitable for a 16-year-old audience [29]. This finding aligns with other studies demonstrating that targeted prompting strategies enabled GPT-4 to generate lay summaries and rewrite complex materials into simpler formats without compromising accuracy. This underscores that employing alternative prompting strategies, such as adding more descriptive context or explicitly targeting lower reading levels, may be crucial for optimizing readability outcomes [2,12]. Such approaches are particularly relevant for patient-facing oncology materials and conversational agents, where clarity and accessibility are critical for informed decision-making [11,30].

This study has limitations. Most importantly, only objective readability measures and participants’ perceived subjective readability were assessed. The study design did not account for specific knowledge retention. Because conversations were open-ended and covered diverse topics, it was not feasible to develop a standardized knowledge test. Our analysis is limited to GPT-4, acknowledging that different LLMs might vary in complexity and output readability. Nevertheless, ChatGPT powered by GPT-4 is among the most widely adopted proprietary models in clinical and public use. Additionally, there is a chance for selection bias by including only patients who agreed to participate. Individuals who declined may systematically differ in their attitudes toward AI due to higher levels of skepticism or rejection. In the present study, this potential selection bias could inflate perceived readability [31]. Another limitation is that participants were recruited during their pre-admission consultations for planned urological interventions. As many participants presented with urooncological conditions, many had recently received a diagnosis and may have used the Internet more intensively. This selection may have contributed to the high perceived readability observed in our study.

Going forward, longitudinal studies incorporating objective knowledge tests, cross-model comparisons, and more diverse participant groups will be essential to fully understand the effect of AI chatbots in patient education.

## 5. Conclusions

In conclusion, this study assessed the readability of GPT-4-based chatbot responses in 231 conversations with urological patients. While objective measures showed texts at a high reading level across topics, most patients perceived the information as clear and understandable, reflecting benefits of multimodal delivery, conversational engagement and elaboration. There was no correlation between objective and subjective readability and no demographic effects. Cognitive biases and response tendencies may influence subjective ratings, underscoring the need for objective knowledge assessments. Prompt engineering can improve readability without loss of accuracy. Additionally, future research should evaluate knowledge retention carefully and test alternative models and prompts.

## Figures and Tables

**Table 1:** Survey items assessing patients’ perceived readability of chatbot responses (5-point Likert scale).

Item		Coding (1–5)
H1	I understood the technical information of the provided information well.	1 = strongly disagree … 5 = strongly agree
H2	I understood the content of the provided information well.	1 = strongly disagree … 5 = strongly agree
E2	The chatbot formulated the provided information clearly.	1 = strongly disagree … 5 = strongly agree
E4	The chatbot explained the provided information well.	1 = strongly disagree … 5 = strongly agree

## Data Availability

Due to privacy concerns, the data have not been made publicly available. However, all data collected or analyzed during this study are available from the corresponding author upon reasonable request. Please contact Lasse Maywald at lasse.maywald@umm.de for access to the data.

## Supplementary Materials

The following supporting information can be downloaded at: https://www.mdpi.com/article/10.3390/curroncol32100582/s1, Table S1: Patient characteristics; Table S2: Conversation characteristics; Table S3: Readability Metrics grouped by Disease; Table S4: Readability Metrics grouped by Procedure; Table S5: Readability Metrics grouped by Theme; Table S6: Regression Results; Table S7: Pairwise correlations among readability indices and patient-reported measures; Table S8: Associations of text length with readability indices; Table S9: Associations of readability indices with perceived readability; Table S10: Patient-reported items and coding scheme; Table S11: Internal consistency of the four-item scale; Table S12: Exploratory factor analysis of perceived readability items; Table S13: Factor loadings and explained variance from exploratory factor analysis of perceived readability items.

## Abbreviations

AMA	American Medical Association
ASL	Average Sentence Length
ASW	Average Syllables per Word
CI	Confidence Interval
DRKS	Deutsches Register Klinischer Studien (German Clinical Trials Register)
EFA	Exploratory Factor Analysis
FRE	Flesch Reading Ease
GPT	Generative Pre-trained Transformer
HoLEP	Holmium Laser Enucleation of the Prostate
IPaLLM	Interaction of Patients with Large Language Models
ISCED	International Standard Classification of Education
LLM	Large Language Model
LIX	Lesbarkeitsindex
NA	Not Applicable
NIH	National Institutes of Health
PEM	Patient Education Material
PSA	Prostate-Specific Antigen
RARP	Robotic-Assisted Radical Prostatectomy
SD	Standard Deviation
WSF	Wiener Sachtextformel

## References

[1] Kutner M., Greenberg E., Jin Y., Paulsen C. (2006). The Health Literacy of America’s Adults: Results from the 2003 National Assessment of Adult Literacy. NCES 2006-483.

[2] Rodler S., Maruccia S., Abreu A., Murphy D., Canes D., Loeb S., Malik R.D., Bagrodia A., Cacciamani G.E. (2024). Readability Assessment of Patient Education Materials on Uro-oncological Diseases Using Automated Measures. Eur. Urol. Focus..

[3] Carl N., Schramm F., Haggenmüller S., Kather J.N., Hetz M.J., Wies C., Michel M.S., Wessels F., Brinker T.J. (2024). Large language model use in clinical oncology. npj Precis. Oncol..

[4] Flesch R. (1948). A new readability yardstick. J. Appl. Psychol..

[5] Pruthi A., Nielsen M.E., Raynor M.C., Woods M.E., Wallen E.M., Smith A.B. (2015). Readability of American online patient education materials in urologic oncology: A need for simple communication. Urology.

[6] Sallam M. (2023). ChatGPT Utility in Healthcare Education, Research, and Practice: Systematic Review on the Promising Perspectives and Valid Concerns. Healthcare.

[7] Cacciamani G.E., Dell’Oglio P., Cocci A., Russo G.I., De Castro Abreu A., Gill I.S., Briganti A., Artibani W. (2021). Asking “Dr. Google” for a Second Opinion: The Devil Is in the Details. Eur. Urol. Focus..

[8] Swoboda C.M., Van Hulle J.M., McAlearney A.S., Huerta T.R. (2018). Odds of talking to healthcare providers as the initial source of healthcare information: Updated cross-sectional results from the Health Information National Trends Survey (HINTS). BMC Fam. Pract..

[9] Carl N., Haggenmüller S., Wies C., Nguyen L., Winterstein J.T., Hetz M.J., Mangold M.H., Hartung F.O., Grüne B., Holland-Letz T. (2025). Evaluating interactions of patients with large language models for medical information. BJU Int..

[10] Davis R., Eppler M., Ayo-Ajibola O., Loh-Doyle J.C., Nabhani J., Samplaski M., Gill I., Cacciamani G.E. (2023). Evaluating the Effectiveness of Artificial Intelligence-powered Large Language Models Application in Disseminating Appropriate and Readable Health Information in Urology. J. Urol..

[11] Hershenhouse J.S., Mokhtar D., Eppler M.B., Rodler S., Storino Ramacciotti L., Ganjavi C., Hom B., Davis R.J., Tran J., Russo G.I. (2025). Accuracy, readability, and understandability of large language models for prostate cancer information to the public. Prostate Cancer Prostatic Dis..

[12] Ganjavi C., Layne E., Cei F., Gill K., Magoulianitis V., Abreu A., Goldenberg M., Desai M.M., Gill I., Cacciamani G.E. (2025). Enhancing Readability of Lay Abstracts and Summaries for Urologic Oncology Literature Using Generative Artificial Intelligence: BRIDGE-AI 6 Randomized Controlled Trial. JCO Clin. Cancer Inform..

[13] Rodler S., Cei F., Ganjavi C., Checcucci E., De Backer P., Rivero Belenchon I., Taratkin M., Puliatti S., Veccia A., Piazza P. (2025). GPT-4 generates accurate and readable patient education materials aligned with current oncological guidelines: A randomized assessment. PLoS ONE.

[14] Carl N., Haggenmüller S., Winterstein J.T., Nguyen L., Wies C., Hetz M.J., Mangold M.H., Grüne B., Michel M.S., Brinker T.J. (2025). Patient insights into empathy, compassion and self-disclosure in medical large language models: Results from the IPALLM III study. World J. Urol..

[15] Carl N., Nguyen L., Haggenmüller S., Joachim Hetz M., Theres Winterstein J., Otto Hartung F., Gruene B., Nikolas Kather J., Holland-Letz T., Stephan Michel M. (2024). Comparing Patient’s Confidence in Clinical Capabilities in Urology: Large Language Models Versus Urologists. Eur. Urol. Open Sci..

[16] Björnsson C.H. (1968). Läsbarhet.

[17] Bamberger R., Vanecek E. (1984). Lesen-Verstehen-Lernen-Schreiben: Die Schwierigkeitsstufen von Texten in Deutscher Sprache.

[18] Amstad T. (1978). Wie Verständlich Sind Unsere Zeitungen?.

[19] Clinton-Lisell V. (2023). Does Reading While Listening to Text Improve Comprehension Compared to Reading Only? A Systematic Review and Meta-Analysis. Educ. Res. Theory Pract..

[20] Kruger J., Dunning D. (1999). Unskilled and unaware of it: How difficulties in recognizing one’s own incompetence lead to inflated self-assessments. J. Pers. Soc. Psychol..

[21] Canady B.E., Larzo M. (2023). Overconfidence in Managing Health Concerns: The Dunning-Kruger Effect and Health Literacy. J. Clin. Psychol. Med. Settings.

[22] Cutilli C.C., Bennett I.M. (2009). Understanding the health literacy of America: Results of the National Assessment of Adult Literacy. Orthop. Nurs..

[23] Lee S.Y., Tsai T.I., Tsai Y.W. (2013). Accuracy in self-reported health literacy screening: A difference between men and women in Taiwan. BMJ Open.

[24] Ware J.E. (1978). Effects of acquiescent response set on patient satisfaction ratings. Med. Care.

[25] Dunsch F., Evans D.K., Macis M., Wang Q. (2018). Bias in patient satisfaction surveys: A threat to measuring healthcare quality. BMJ Glob. Health.

[26] Chang E.M., Gillespie E.F., Shaverdian N. (2019). Truthfulness in patient-reported outcomes: Factors affecting patients’ responses and impact on data quality. Patient Relat. Outcome Meas..

[27] Sahin S., Erkmen B., Duymaz Y.K., Bayram F., Tekin A.M., Topsakal V. (2024). Evaluating ChatGPT-4’s performance as a digital health advisor for otosclerosis surgery. Front. Surg..

[28] Stephenson-Moe C.A., Behers B.J., Gibons R.M., Behers B.M., Jesus Herrera L., Anneaud D., Rosario M.A., Wojtas C.N., Bhambrah S., Hamad K.M. (2025). Assessing the quality and readability of patient education materials on chemotherapy cardiotoxicity from artificial intelligence chatbots: An observational cross-sectional study. Medicine.

[29] Thia I., Saluja M. (2024). ChatGPT: Is This Patient Education Tool for Urological Malignancies Readable for the General Population?. Res. Rep. Urol..

[30] King R.C., Samaan J.S., Haquang J., Bharani V., Margolis S., Srinivasan N., Peng Y., Yeo Y.H., Ghashghaei R. (2025). Improving the Readability of Institutional Heart Failure-Related Patient Education Materials Using GPT-4: Observational Study. JMIR Cardio.

[31] Jiang X., Wang L., Leng Y., Xie R., Li C., Nie Z., Liu D., Wang G. (2024). The level of electronic health literacy among older adults: A systematic review and meta-analysis. Arch. Public Health.

