# Supplementary material for: Readability of Chatbot Responses in Prostate Cancer and Urological Care: Objective Metrics Versus Patient Perceptions
Source: Curr Oncol. 2025 Oct 19;32(10):582. doi: 10.3390/curroncol32100582 (PMC12564201; doi:10.3390/curroncol32100582)
Supplement: Supplementary file 1 [file curroncol-32-00582-s001.zip › curroncol-3915078-supplementary.pdf]

Supplementary results

Table S1: Patient characteristics

Patient characteristics by age group. Values are mean (SD) or n/N (%). Group comparisons follow the tests noted in the table footnote.

Characteristic	Overall N = 230[1]	Young Adult (18 - 29 years) N = 20[1]	Adult (30 - 49 years) N = 20[1]	Senior (50 - 65 years) N = 75[1]	Elderly (≥ 65 years) N = 115[1]	p-value[2]
**Age (years)**	60.97 (15.91)	24.45 (2.48)	38.25 (5.18)	59.31 (3.83)	72.37 (6.02)	<0.001
**Number of doctor visits last year**						0.4
1 to 5 times	80 / 229 (35%)	4 / 20 (20%)	8 / 20 (40%)	30 / 74 (41%)	38 / 115 (33%)	
more than 5 times	148 / 229 (65%)	16 / 20 (80%)	12 / 20 (60%)	43 / 74 (58%)	77 / 115 (67%)	
not at all	1 / 229 (0.4%)	0 / 20 (0%)	0 / 20 (0%)	1 / 74 (1.4%)	0 / 115 (0%)	
NA	1	0	0	1	0	
**Prior use of internet for medical information**						0.2
yes	195 / 229 (85%)	19 / 20 (95%)	19 / 20 (95%)	65 / 74 (88%)	92 / 115 (80%)	
no	34 / 229 (15%)	1 / 20 (5.0%)	1 / 20 (5.0%)	9 / 74 (12%)	23 / 115 (20%)	
NA	1	0	0	1	0	
**Frequency of internet use for medical information**						0.7
daily	119 / 229 (52%)	12 / 20 (60%)	12 / 20 (60%)	38 / 74 (51%)	57 / 115 (50%)	
monthly	34 / 229 (15%)	2 / 20 (10%)	1 / 20 (5.0%)	9 / 74 (12%)	22 / 115 (19%)	
weekly	76 / 229 (33%)	6 / 20 (30%)	7 / 20 (35%)	27 / 74 (36%)	36 / 115 (31%)	

Characteristic	Overall<br>N = 230[1]	Young Adult<br>(18 - 29 years)<br>N = 20[1]	Adult<br>(30 - 49 years)<br>N = 20[1]	Senior<br>(50 - 65 years)<br>N = 75[1]	Elderly<br>(≥ 65 years)<br>N = 115[1]	p-value[2]
NA	1	0	0	1	0	
**Internet Source for medical information**						0.8
Bing	3 / 214 (1.4%)	0 / 18 (0%)	0 / 19 (0%)	0 / 71 (0%)	3 / 106 (2.8%)	
Ecosia	1 / 214 (0.5%)	0 / 18 (0%)	0 / 19 (0%)	1 / 71 (1.4%)	0 / 106 (0%)	
Google	181 / 214 (85%)	16 / 18 (89%)	16 / 19 (84%)	61 / 71 (86%)	88 / 106 (83%)	
Health insurance	1 / 214 (0.5%)	0 / 18 (0%)	0 / 19 (0%)	0 / 71 (0%)	1 / 106 (0.9%)	
In Official patient forum	1 / 214 (0.5%)	0 / 18 (0%)	0 / 19 (0%)	1 / 71 (1.4%)	0 / 106 (0%)	
Medical research database	5 / 214 (2.3%)	1 / 18 (5.6%)	2 / 19 (11%)	0 / 71 (0%)	2 / 106 (1.9%)	
Official patient forum	8 / 214 (3.7%)	1 / 18 (5.6%)	0 / 19 (0%)	3 / 71 (4.2%)	4 / 106 (3.8%)	
Pharmaceutical	3 / 214 (1.4%)	0 / 18 (0%)	0 / 19 (0%)	1 / 71 (1.4%)	2 / 106 (1.9%)	
Wikipedia	8 / 214 (3.7%)	0 / 18 (0%)	1 / 19 (5.3%)	3 / 71 (4.2%)	4 / 106 (3.8%)	
Youtube	3 / 214 (1.4%)	0 / 18 (0%)	0 / 19 (0%)	1 / 71 (1.4%)	2 / 106 (1.9%)	
NA	16	2	1	4	9	
**Prior use of LLM for medical information**						0.029
ja	20 / 222 (9.0%)	3 / 20 (15%)	5 / 20 (25%)	6 / 71 (8.5%)	6 / 111 (5.4%)	
Nein	202 / 222 (91%)	17 / 20 (85%)	15 / 20 (75%)	65 / 71 (92%)	105 / 111 (95%)	
NA	8	0	0	4	4	

Characteristic	Overall N = 230[1]	Young Adult (18 - 29 years) N = 20[1]	Adult (30 - 49 years) N = 20[1]	Senior (50 - 65 years) N = 75[1]	Elderly (≥ 65 years) N = 115[1]	p-value[2]
**Which LLM**						0.11
ChatGPT	19 / 22 (86%)	5 / 5 (100%)	3 / 5 (60%)	5 / 6 (83%)	6 / 6 (100%)	
Copilot	1 / 22 (4.5%)	0 / 5 (0%)	0 / 5 (0%)	1 / 6 (17%)	0 / 6 (0%)	
Gemini	2 / 22 (9.1%)	0 / 5 (0%)	2 / 5 (40%)	0 / 6 (0%)	0 / 6 (0%)	
NA	208	15	15	69	109	
**Sex**						0.004
m	167 / 226 (74%)	9 / 20 (45%)	14 / 20 (70%)	52 / 74 (70%)	92 / 112 (82%)	
w	59 / 226 (26%)	11 / 20 (55%)	6 / 20 (30%)	22 / 74 (30%)	20 / 112 (18%)	
NA	4	0	0	1	3	
**Educational lever in accordance with International Standard Classification of Education (ISCED)**						0.3
1	26 / 193 (13%)	0 / 16 (0%)	2 / 18 (11%)	11 / 64 (17%)	13 / 95 (14%)	
2	68 / 193 (35%)	8 / 16 (50%)	7 / 18 (39%)	20 / 64 (31%)	33 / 95 (35%)	
3	31 / 193 (16%)	7 / 16 (44%)	3 / 18 (17%)	10 / 64 (16%)	11 / 95 (12%)	
4	68 / 193 (35%)	1 / 16 (6.3%)	6 / 18 (33%)	23 / 64 (36%)	38 / 95 (40%)	
NA	37	4	2	11	20	

[1]Mean (SD); n / N (%) [2]Kruskal-Wallis rank sum test; Fisher's exact test; Pearson's Chi-squared test depending on data type and number of categories

Table S2: Conversation characteristics

Conversation characteristics: number of questions per interaction, number of sources, and source ratio. Values are n/N (%) or mean (SD).

Descriptive Summary	
**Characteristic**	**N = 231**[1]
**Number of Questions**	
1	138 / 231 (60%)
2	74 / 231 (32%)
3	13 / 231 (5.6%)
4	4 / 231 (1.7%)
5	1 / 231 (0.4%)
6	1 / 231 (0.4%)
**Number of Sources**	6.84 (4.03)
**Source Ratio**	4.47 (1.36)

[1]n / N (%); Mean (SD)

Table S3 Readability Metrics grouped by Disease

Readability metrics by disease category. Flesch Reading Ease, LIX, and Wiener Sachtextformel with Kruskal–Wallis p-values, epsilon-squared effect sizes, confidence intervals, and magnitude labels; length features shown for context.

Descriptive Statistics

Characteristic	Overall N = 231[1]	Prostate cancer N = 52[1]	Bladder cancer N = 32[1]	PSA-elevation N = 27[1]	BPS N = 25[1]	NA* N = 22[1]	Gender-dysphoria N = 18[1]	Kidney cancer N = 17[1]	Urolithiasis N = 16[1]	Functional kidney disease N = 14[1]	Urethral stricture N = 4[1]	Testicular cancer N = 2[1]	Penile cancer N = 1[1]	UTUC N = 1[1]	p-value[2] Effect size(ε^2) 95%CI Magnitude
Flesch Reading Ease	43.07 (9.06)	42.11 (9.24)	42.50 (9.70)	44.84 (7.09)	46.68 (8.55)	44.69 (9.58)	38.55 (10.75)	40.99 (8.28)	44.51 (7.02)	39.82 (8.29)	46.02 (9.07)	56.08 (7.02)	33.92 (NA)	48.52 (NA)	p=0.071 (ε^2)=0.036 CI=0.005–0.139 small
LIX Readability	52.82 (6.17)	53.73 (6.78)	52.18 (5.37)	51.82 (6.17)	50.35 (6.08)	52.69 (6.15)	56.86 (6.20)	51.78 (6.01)	53.62 (4.50)	54.19 (4.84)	50.31 (6.46)	43.71 (8.04)	58.26 (NA)	52.71 (NA)	p=0.071 (ε^2)=0.036 CI=0.006–0.143 small
WSF 1	11.20 (1.63)	11.33 (1.71)	11.26 (1.62)	10.95 (1.39)	10.57 (1.75)	11.03 (1.56)	12.15 (1.80)	11.01 (1.70)	11.32 (1.15)	11.73 (1.14)	10.72 (1.85)	8.37 (2.25)	13.42 (NA)	10.87 (NA)	p=0.10 (ε^2)=0.030 CI=0.003–0.128 small
Number of Words	175.04 (54.20)	169.98 (49.86)	169.01 (55.34)	194.81 (57.37)	178.88 (52.28)	173.28 (60.74)	176.91 (50.46)	181.27 (65.02)	167.66 (40.83)	166.80 (67.71)	195.75 (38.57)	127.65 (49.00)	154.00 (NA)	167.00 (NA)	0.7
Number of Sentences	14.74 (6.52)	13.72 (6.57)	15.80 (7.72)	16.11 (6.79)	17.49 (6.43)	14.80 (6.41)	13.44 (5.52)	15.47 (6.77)	11.88 (4.16)	13.51 (5.67)	15.75 (6.08)	10.35 (4.74)	10.00 (NA)	11.00 (NA)	0.2

[1]Mean (SD) [2]Kruskal-Wallis rank sum test

NA**NA because only Procedure-related questions and not disease questions

Table S4 Readability Metrics grouped by Procedure

Readability metrics by procedure category. Flesch Reading Ease, LIX, and Wiener Sachtextformel with Kruskal–Wallis p-values, effect sizes, and confidence intervals; length features shown for context.

Descriptive Statistics

Characteristic	Overall N=231[1]	RARP N=46[1]	NA* N=44[1]	Prostate biopsy N= 27[1]	HoLEP N=24[1]	Trans-gender N=18[1]	NX/ NTUX N=17[1]	Litho-trypsie N=13[1]	TURBT N=13[1]	CX and UD N=12[1]	DJ N=9[1]	Urethral reconstruction N=3[1]	Chemo-therapy N=2[1]	Orchidec-tomy N=1[1]	partial Penect-omy N=1[1]	Pyelo-plasty N=1[1]	p-value[2] Effect size(ε^2) 95%CI Magnitude
Flesch Reading Ease	43.07 (9.06)	41.43 (9.06)	43.67 (9.42)	44.84 (7.09)	46.64 (8.73)	38.55 (10.75)	41.22 (8.32)	44.16 (8.11)	43.97 (10.69)	41.43 (8.37)	42.82 (8.02)	49.43 (7.33)	42.50 (12.19)	61.04 (NA)	33.92 (NA)	40.66 (NA)	p=0.2 (ε^2)=0.01 CI=-0.014–0.124) small
LIX Readability	52.82 (6.17)	54.27 (6.64)	52.61 (6.35)	51.82 (6.17)	50.62 (6.06)	56.86 (6.20)	51.08 (5.64)	54.18 (3.78)	51.76 (6.07)	52.85 (4.98)	53.54 (3.69)	47.14 (1.52)	52.97 (5.06)	38.02 (NA)	58.26 (NA)	47.21 (NA)	p=0.031 (ε^2)=0.05 CI=0.006–0.147 small
WSF 1	11.20 (1.63)	11.41 (1.65)	11.24 (1.64)	10.95 (1.39)	10.61 (1.77)	12.15 (1.80)	10.93 (1.69)	11.39 (1.09)	11.04 (1.78)	11.35 (1.37)	11.36 (1.38)	9.85 (0.79)	11.23 (1.79)	6.78 (NA)	13.42 (NA)	10.94 (NA)	p=0.2 (ε^2)=0.021 CI=-0.013–0.113 small
Number of Words	175.04 (54.20)	171.49 (50.46)	168.47 (47.66)	194.81 (57.37)	177.46 (52.91)	176.91 (50.46)	177.04 (64.19)	164.58 (33.94)	137.90 (49.75)	195.01 (41.80)	195.05 (93.08)	205.33 (40.99)	219.65 (81.11)	93.00 (NA)	154.00 (NA)	90.50 (NA)	0.10
Number of Sentences	14.74 (6.52)	13.30 (6.57)	15.11 (6.43)	16.11 (6.79)	16.93 (5.90)	13.44 (5.52)	15.76 (6.67)	11.27 (3.23)	12.32 (6.83)	18.32 (8.48)	14.93 (7.48)	17.67 (5.77)	15.85 (3.04)	7.00 (NA)	10.00 (NA)	15.50 (NA)	0.090

[1]Mean (SD) [2]Kruskal-Wallis rank sum test

*NA because only Disease-related questions and not procedure questions

Table S5 Readability Metrics grouped by Theme

Readability metrics by conversation theme. Flesch Reading Ease, LIX, and Wiener Sachtextformel with Kruskal–Wallis p-values, effect sizes, and confidence intervals; length features shown for context.

Descriptive Statistics

Characteristic	Overall N = 231[1]	Diagnostic N = 6[1]	Disease N = 39[1]	Therapy N = 35[1]	Prevention N = 4[1]	Medication N = 3[1]	Follow-Up N = 43[1]	Prognosis N = 19[1]	Complications N = 19[1]	Behaviour N = 6[1]	Procedural N = 39[1]	Psychosocial N = 2[1]	Other N = 16[1]	p-value[2] Effect size(ε²) 95%CI Magnitude
**Flesch Reading Ease**	43.07 (9.06)	43.23 (8.96)	43.50 (9.60)	42.43 (11.43)	45.07 (8.47)	36.97 (9.96)	44.04 (8.29)	42.76 (8.22)	43.84 (7.68)	44.72 (9.01)	41.44 (7.33)	38.65 (17.27)	44.79 (10.97)	p>0.9 (ε²)=-0.32 CI=-0.024–0.082 small
**LIX Readability**	52.82 (6.17)	55.34 (7.87)	53.92 (5.43)	51.16 (7.23)	53.99 (1.87)	54.93 (6.14)	51.33 (5.07)	52.80 (5.18)	53.09 (6.41)	51.52 (4.90)	53.79 (6.32)	56.17 (12.03)	53.53 (8.14)	p=0.24 (ε²)=0.013 CI=0.002–0.148 small
**WSF 1**	11.20 (1.63)	11.41 (1.53)	11.33 (1.56)	11.24 (2.07)	11.55 (0.87)	12.41 (1.36)	10.69 (1.53)	11.09 (1.54)	11.14 (1.43)	10.95 (1.24)	11.58 (1.43)	12.30 (3.17)	11.01 (1.96)	p=0.6 (ε²)=0.009 CI=-0.012–0.106 small
**Number of Words**	175.04 (54.20)	149.50 (30.48)	159.35 (52.22)	166.21 (61.34)	177.75 (26.30)	165.83 (22.15)	199.70 (59.99)	172.40 (47.14)	163.27 (41.83)	192.67 (43.31)	186.54 (57.15)	181.13 (26.69)	158.69 (38.83)	0.007
**Number of Sentences**	14.74 (6.52)	12.33 (9.61)	12.10 (5.41)	18.45 (7.97)	12.75 (2.50)	14.83 (3.55)	16.60 (6.19)	13.18 (4.53)	13.34 (7.13)	15.42 (3.93)	14.74 (6.19)	17.25 (7.42)	12.44 (4.94)	0.004

[1]Mean (SD) [2]Kruskal-Wallis rank sum test

*NA because only Disease-related questions and not procedure questions

Table S6 Regression Results

Regression results for education level and age on perceived readability items (H1, H2, E2, E4) and composite readability index. Reported as beta, standard error, test statistic, p-value, and 95% confidence interval.

Term	(β)	std.error	statistic	P value	95%CI (lower)	95%CI (upper)	Outcome variable
Education	0.061	0.043	1.386	0.167	-0.025	0.146	H1
Education	0.052	0.051	1.053	0.293	-0.046	0.151	H2
Education	0.003	0.048	0.061	0.951	-0.093	0.099	E2
Education	0.022	0.061	0.372	0.711	-0.097	0.142	E4
Education	0.186	0.156	1.188	0.236	-0.123	0.495	read_index
Age	-0.003	0.003	-1.174	0.241	-0.009	0.002	H1
Age	-0.005	0.003	-1.429	0.154	-0.011	0.001	H2
Age	-0.003	0.003	-1.011	0.313	-0.009	0.003	E2
Age	0.001	0.004	0.145	0.884	-0.007	0.008	E4
Age	-0.012	0.011	-1.174	0.241	-0.033	0.008	read_index

Table S7 Pairwise correlations among readability indices and patient-reported measures.
Spearman ρ with 95% confidence intervals and p-values; qualitative interpretation of strength is provided.

**Category**	**Pair**	ρ **(95% CI)**	**p-value**	**Interpretation**
Readability index ↔ Readability index	FRE vs WSF-1	−0.917 [−0.927, −0.872]	<0.001	Strong inverse
	FRE vs LIX	−0.802 [−0.850, −0.742]	<0.001	Strong inverse
	LIX vs WSF-1	0.872 [0.828, 0.903]	<0.001	Strong positive
PROMs & composite	H1 vs H2	0.816 [0.751, 0.876]	<0.001	Strong
	H1 vs read_index	0.756 [0.694, 0.811]	<0.001	Strong
	H2 vs read_index	0.743 [0.673, 0.803]	<0.001	Strong
	E4 vs read_index	0.751 [0.677, 0.810]	<0.001	Strong
	E2 vs read_index	0.726 [0.646, 0.789]	<0.001	Strong
	E2 vs E4	0.654 [0.561, 0.738]	<0.001	Moderate-strong

Table S7 cont'd Pairwise correlations among readability indices and patient-reported measures.
Spearman ρ with 95% confidence intervals and p-values; qualitative interpretation of strength is provided.

Category	Pair	ρ (95% CI)	p-value	Interpretation
Readability index ↔ Readability index	FRE vs WSF-1	−0.917 [−0.927, −0.872]	<0.001	Strong inverse
	FRE vs LIX	−0.802 [−0.850, −0.742]	<0.001	Strong inverse
	LIX vs WSF-1	0.872 [0.828, 0.903]	<0.001	Strong positive
PROMs & composite	H1 vs H2	0.816 [0.751, 0.876]	<0.001	Strong
	H1 vs read_index	0.756 [0.694, 0.811]	<0.001	Strong
	H2 vs read_index	0.743 [0.673, 0.803]	<0.001	Strong
	E4 vs read_index	0.751 [0.677, 0.810]	<0.001	Strong
	E2 vs read_index	0.726 [0.646, 0.789]	<0.001	Strong
	E2 vs E4	0.654 [0.561, 0.738]	<0.001	Moderate-strong

Table S7 cont'd Pairwise correlations among readability indices and patient-reported measures.
Spearman ρ with 95% confidence intervals and p-values; qualitative interpretation of strength is provided

Category	Pair	ρ (95% CI)	p-value	Interpretation
Readability index ↔ Readability index	FRE vs WSF-1	−0.917 [−0.927, −0.872]	<0.001	Strong inverse
	FRE vs LIX	−0.802 [−0.850, −0.742]	<0.001	Strong inverse
	LIX vs WSF-1	0.872 [0.828, 0.903]	<0.001	Strong positive
PROMs & composite	H1 vs H2	0.816 [0.751, 0.876]	<0.001	Strong
	H1 vs read_index	0.756 [0.694, 0.811]	<0.001	Strong
	H2 vs read_index	0.743 [0.673, 0.803]	<0.001	Strong
	E4 vs read_index	0.751 [0.677, 0.810]	<0.001	Strong
	E2 vs read_index	0.726 [0.646, 0.789]	<0.001	Strong
	E2 vs E4	0.654 [0.561, 0.738]	<0.001	Moderate-strong

Table S8 Associations of text length with readability indices.
Spearman ρ, as indicated, with 95% confidence intervals, p-values, and brief interpretation.

**Pairs**	**ρ (95% CI)**	**p-value**	**Interpretation**
n_words vs n_sentences	0.573 [0.446, 0.647]	<0.001	Moderate (longer texts → more sentences)
LIX vs n_sentences	−0.455 [−0.562, −0.334]	<0.001	Moderate inverse
**Pairs**	**ρ (95% CI)**	**p-value**	**Interpretation**
FRE vs n_sentences	0.198 [0.072, 0.321]	0.003	Weak
FRE vs n_words	−0.169 [−0.291, −0.044]	0.010	Weak
WSF-1 vs n_sentences	−0.211 [−0.338, −0.076]	0.001	Weak
LIX vs n_words	0.140 [0.010, 0.264]	0.033	Weak

Table S9 Associations of readability indices wit perceived readability.
Spearman ρ, as indicated, with 95% confidence intervals, p-values, and brief interpretation.

Group	Summary
Readability indeces ↔ perceived readability	FRE with E2/E4/H1/H2: $\rho \approx -0.005$ to 0.096, $p = 0.146–0.939$ (ns)
	LIX with E2/E4/H1/H2: $\rho \approx -0.080$ to 0.046, $p = 0.226–0.974$ (ns)
	WSF-1 with E2/E4/H1/H2: $\rho \approx -0.034$ to 0.018, $p = 0.605–0.969$ (ns)
Readability index (FRE) ↔ perceived read_index	FRE $\rho = 0.026$, LIX $\rho = 0.015$, WSF-1 $\rho = 0.018$ — all non-significant

Table S10 Patient-reported items and coding scheme.

English and original German wording for H1, H2, E2, and E4 with 5-point Likert coding.

Item	English Wording	Original German	Coding (1–5)
**H1**	I understood the technical information of the provided information well.	Ich habe die gelieferten Informationen sprachlich gut verstanden.	1 = strongly disagree ... 5 = strongly agree
**H2**	I understood the content of the provided information well.	Ich habe die gelieferten Informationen inhaltlich gut verstanden.	1 = strongly disagree ... 5 = strongly agree
**E2**	The chatbot formulated the provided information clearly.	Das Sprachmodell hat die gelieferten Informationen klar formuliert.	1 = strongly disagree ... 5 = strongly agree
**E4**	The chatbot explained the provided information well.	Das Sprachmodell hat die gelieferten Informationen gut erklärt.	1 = strongly disagree ... 5 = strongly agree

Table S11 Internal consistency of the four-item scale.

Cronbach’s alpha with 95% confidence interval, standardized alpha, and average inter-item correlation.

Number of Items	Cronbach.s Alpha	95%CI	Average Inter-item Correlation	Standardized Alpha
4	0.75	0.69 – 0.8	0.43	0.75

Table S12 Exploratory factor analysis of perceived readability items.
Factor loadings, communalities, uniqueness, and variance explained for a two-factor solution.

Item	Factor 1 (ML1)	Factor 2 (ML2)	Communality (h^2)	Uniqueness (u^2)	Notes
**H1**	0.76	0.08	0.62	0.38	Strong loading on F1
**H2**	1.01	-0.03	1.00	0.01	Extremely strong loading on F1
**E2**	-0.03	1.01	1.00	0.01	Extremely strong loading on F2
**E4**	0.11	0.64	0.46	0.54	Moderate loading on F2

Table S13 Factor loadings and explained variance from exploratory factor analysis of perceived readability items.

Factor	SS Loadings	Proportion Variance	Cumulative Variance
**Factor 1 (ML1)**	1.63	0.41 (41%)	0.41 (41%)
**Factor 2 (ML2)**	1.45	0.36 (36%)	0.77 (77%)
